# Case report of a giant hypervascular mediastinal mass with airway and SVC compromise managed without preoperative biopsy

**DOI:** 10.3389/fonc.2026.1871556

**Published:** 2026-06-22

**Authors:** Rongjuan Jiang, Shijin Ma, Ke Gao, Fengjuan Lei, Xiaohui Cao, Shaoxing Liu

**Affiliations:** 1 Department of Anesthesiology, West China School of Medicine, Sichuan University, Sichuan University Affiliated Chengdu Second People’s Hospital, Chengdu Second People’s Hospital, Chengdu, Sichuan, China; 2 Department of Cardiothoracic Surgery, West China School of Medicine, Sichuan University, Sichuan University Affiliated Chengdu Second People’s Hospital, Chengdu Second People’s Hospital, Chengdu, Sichuan, China; 3 Department of General Electric Medical Systems, General Electric (GE) Medical Systems Trade and Development (Shanghai) Limited Company, Chengdu, Sichuan, China

**Keywords:** Castleman disease, follicular dendritic cell sarcoma, mediastinal tumor, multidisciplinary management, superior vena cava syndrome, tracheal stenosis

## Abstract

**Rationale:**

To summarize the clinical experience of multidisciplinary management for giant hypervascular mediastinal follicular dendritic cell sarcoma.

**Patient concerns:**

A 37-year-old man had a one-week history of progressive dyspnea, orthopnea, and morning facial swelling.

**Diagnoses:**

Preoperative imaging suggested Castleman disease as the leading differential diagnosis, and pathological examination confirmed follicular dendritic cell sarcoma (FDCS).

**Interventions:**

The patient received an effective strategy under the supervision of multidisciplinary specialists, including 3D reconstruction for precise surgical planning, preoperative angioembolization to reduce vascularity, awake fiberoptic intubation, and video-assisted thoracoscopic surgery (VATS).

**Learning points:**

In hypervascular mediastinal tumors, angioembolization of feeding vessels performed on the day of surgery maximally reduces intraoperative bleeding while avoiding complications of prolonged devascularization. When tracheal stenosis and SVC syndrome coexist, a sequential positioning strategy is essential: tumor-side tilt for awake intubation, followed by left lateral decubitus to relieve SVC compression. When biopsy risk is prohibitive, upfront surgical resection offers simultaneous decompression and definitive diagnosis after multidisciplinary discussion.

## 1 Introduction

Follicular dendritic cell sarcoma (FDCS) is a rare sarcoma of mesenchymal dendritic cell origin, accounting for <0.4% of all soft tissue sarcomas (1, 2). It can arise within lymph nodes (nodal type) or at extranodal sites, including the gastrointestinal tract, uterus, and spleen, with nonspecific clinical presentations (2, 3). However, mediastinal FDCS is exceptionally uncommon, with very few primary cases described to date. In contrast, unicentric Castleman disease (UCD) is a critical differential diagnosis for anterior or middle mediastinal masses (4). Both entities may present as large soft tissue lesions with intense ¹^8^F-FDG uptake on PET/CT and regional lymph node involvement, leading to substantial overlap in clinical and imaging features (3, 5). This diagnostic ambiguity complicates preoperative assessment and often requires histopathology, immunohistochemical profiling (e.g. CD21, CD23 as follicular dendritic cell markers), for definitive distinction (3).

Here, we report a challenging case of a giant mediastinal tumor presenting with life-threatening airway and superior vena cava (SVC) compression. Although the mass was initially suspected to be unicentric Castleman disease (UCD) based on preoperative contrast-enhanced computed tomography, postoperative pathological evaluation confirmed a definitive diagnosis of primary follicular dendritic cell sarcoma (FDCS), with no background Castleman-like features observed. The tumor was successfully managed using a meticulously planned multidisciplinary approach. This case may provide a useful reference for the management of similarly high-risk mediastinal tumors.

## 2 Case report

### 2.1 Case presentation

A 37-year-old man was referred to our hospital for evaluation of a large superior mediastinal mass and surgical intervention.

Seven days earlier, he had presented to a local clinic with progressive dyspnea, orthopnea, and morning facial swelling. Radiographic examination at that time revealed a large mass in the right superior mediastinum, prompting referral for further management. The patient denied any history of diabetes mellitus, chronic pulmonary disease, asthma, chronic heart disease, or other relevant comorbidities. He reported smoking approximately 10 cigarettes per day and had no other significant personal or family medical history. He had not received any treatment before admission.

### 2.2 Physical examination findings

At admission, the patient’s vital signs were as follows: temperature, 36.3 °C; blood pressure 127/78 mm Hg, heart rate 62 beats/min; respiratory rate 17 breaths/min, oxygen saturation 96% on room air, and body mass index (BMI) 27.97 kg/m². Because of dyspnea and orthopnea, physical examination was performed with the patient in a sitting position. Cardiac examination revealed a regular rhythm without additional heart sounds or murmurs. Marked jugular venous distension was observed. Pulmonary auscultation demonstrated clear breath sounds without rales. No peripheral edema or lymphadenopathy was noted, and there were no cutaneous or articular abnormalities. Abdominal and neurologic examinations were unremarkable.

### 2.3 Diagnostic studies

Laboratory workup revealed a hemoglobin level of 13.2 g/dL and a platelet count of 103 × 10³/μL along with normal hepatic and renal biochemical functions. To assess pulmonary function, pulmonary function testing demonstrated severe intrathoracic airway obstruction without bronchodilator response; all other measured pulmonary parameters were within normal limits. Contrast-enhanced computed tomography (CT) suggested unicentric Castleman disease (UCD) based on specific imaging features, including a solid mass with well-circumscribed borders, marked heterogeneous enhancement, internal nodular non-enhancing low-density areas, and abundant surrounding feeding vessels. Three-dimensional (3D) reconstruction imaging further demonstrated displacement and marked compression of both the superior vena cava (SVC) and trachea by the mediastinal mass, which measured approximately 9 × 9 × 9 cm. The tracheal lumen was severely narrowed, with the narrowest diameter measuring approximately 4 mm (**Figure 1**). 3D reconstruction of the tumor and its relationship to adjacent structures is provided (**Supplementary Video 1**). Digital subtraction angiography (DSA) revealed abundant tumor vascularity, with the thyrocervical trunk (a branch of the subclavian artery), right internal thoracic artery, and right intercostal arteries identified as the main feeding vessels (**Figure 2**). Abdominal ultrasonography revealed no evidence of additional masses.

**Figure 1:**
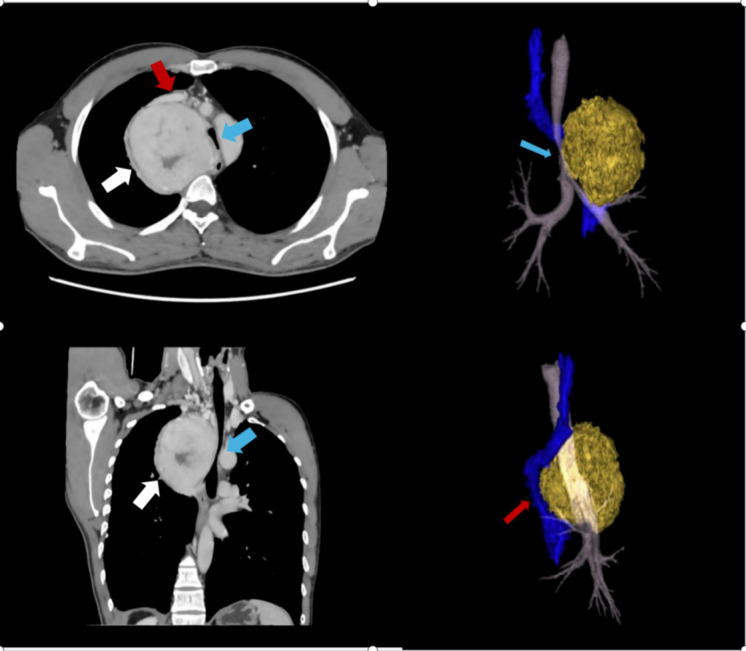
Contrast-enhanced chest CT and 3D reconstruction showing a large right mediastinal mass compressing the trachea and superior vena cava. In the CT images (left), the white arrows indicate the huge mediastinal mass, the blue arrows point to the compressed airway (trachea), and the red arrows highlight the SVC. In the 3D reconstruction models (right), the anatomical structures are color-coded to clearly demonstrate their spatial relationships: the mass is depicted in yellow, the SVC in blue, and the trachea in gray.

**Figure 2:**
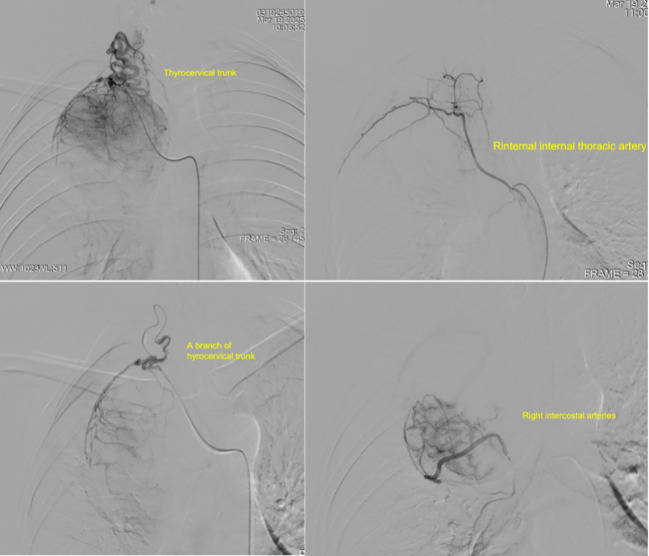
Superselective arterial embolization of the tumor’s feeding vessels under digital subtraction angiography (DSA).

### 2.4 Clinical course

The patient elected to undergo surgery. To minimize intraoperative bleeding, CTA-guided embolization of the tumor-feeding vessels was performed on the day of surgery. *To* guarantee perioperative safety, a sequential positioning strategy was carefully implemented. Initially, the patient was placed in a right semi-recumbent position (45°) for awake fiberoptic endotracheal intubation. Immediately following the successful establishment of the artificial airway, the patient was transitioned to a left-tilted position for anesthesia induction. Finally, the patient was turned to the left lateral decubitus position to achieve optimal surgical exposure and further relieve mechanical compression on the right superior vena cava (SVC). Video-assisted thoracoscopic surgery was performed. Dense thoracic adhesions were encountered, and a moderate amount of pleural effusion was present. The SVC was carefully dissected and suspended to maximize exposure and avoid injury (**Figure 3**). The tumor, measuring approximately 9 × 9 × 9 cm, was successfully excised. On gross inspection, the tumor was predominantly solid, with a tan-yellow cut surface (**Figure 3**). Owing to preoperative embolization of the feeding arteries, estimated intraoperative blood loss was only 20 mL, with a total infusion volume of 1000 mL. The patient awoke from anesthesia without complications, with immediate resolution of dyspnea, and was able to ambulate within 3 hours. He was discharged on the 3^rd^ postoperative day without adjuvant therapy. Eventually, he was diagnosed with follicular dendritic cell sarcoma (FDCS) by histopathologic examination. Microscopically, the tumor was composed of spindle cells arranged in fascicles and solid sheets. The neoplastic cells exhibited clear cytologic atypia, although mitotic activity was infrequent. A prominent mixed lymphoplasmacytic background (comprising infiltrating B and T lymphocytes) along with fibroblastic proliferation was observed throughout the stroma. Focal areas of necrosis were present, and these were considered secondary to the preoperative transcatheter embolization. The surgical margins were clear of tumor involvement. Immunohistochemical (IHC) staining revealed that the tumor cells were positive for CD21 and CD23, and the Ki-67 proliferative index was low. Additionally, the tumor cells were negative for CD20, CD3, CD138, Pan-CK, and EBER-ISH. Based on these comprehensive morphological and immunophenotypic profiles, a definitive diagnosis of primary mediastinal follicular dendritic cell sarcoma (FDCS) was established (**Figure 4**). The comprehensive immunohistochemical panel is summarized in **Supplementary Table 1**.

**Figure 3:**
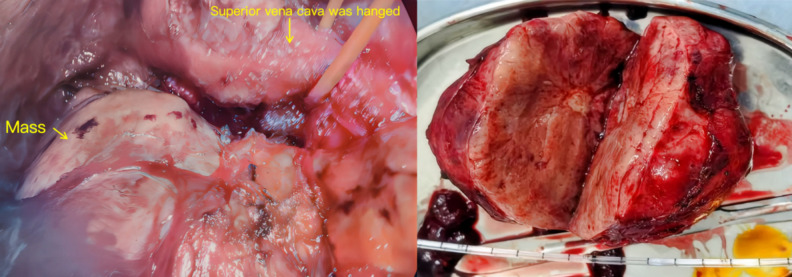
Thoracoscopic view showing the tumor located beneath and compressing the SVC (left).The cross-section revealed solid areas (right).

**Figure 4:**
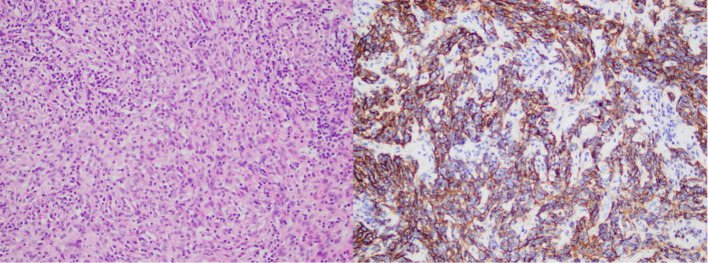
Histopathologic findings. **(A)** Hematoxylin and eosin-stained section showing spindle cells arranged in fascicles and sheets. **(B)** Immunohistochemical staining showing strong and diffuse CD21 positivity, supporting follicular dendritic cell differentiation.

A routine follow-up complete blood count (CBC) before discharge demonstrated that the platelet count had already spontaneously normalized to 156 × 10³/μL. Based on the completely negative surgical margins, the low-grade morphological features of the tumor, and the patient’s rapid postoperative symptomatic improvement, the multidisciplinary team (MDT) recommended no further adjuvant therapy. Consequently, a strategy of close clinical and radiological surveillance was adopted, which also aligned with the patient’s preference. Then, the patient was discharged on postoperative day 3. At 5 months postoperatively, the patient reported being asymptomatic via telephone contact, but he refused further hospital visits. Unfortunately, at 6 months postoperatively, the patient could not be reached by telephone and was lost to follow-up. Consequently, no long-term imaging or laboratory data are available after this point.

## 3 Discussion

This case describes a large mediastinal mass caused by FDCS, complicated by superior vena cava (SVC) syndrome and severe airway narrowing. It is notable for the combination of large tumor size, marked hypervascularity, severe tracheal narrowing, and SVC compression. Complete surgical resection resulted in immediate symptom relief and a favorable short-term outcome, underscoring the clinical and educational importance of this presentation.

FDCS is a rare malignant neoplasm of follicular dendritic cell origin with variable clinical behavior (6). Due to the rarity of this disease, no standard treatment protocol exists. For localized FDCS, complete surgical resection is the preferred and most commonly recommended approach, with or without adjuvant therapy (7). Notably, a review of the literature indicates that most previously reported mediastinal FDCS cases are asymptomatic or present with mild, non-specific chest pain (8, 9). In contrast, our case was complicated by acute, life-threatening compression of both the SVC and trachea, which substantially increased the clinical complexity. Differentiating FDCS from unicentric Castleman disease (UCD) can be challenging due to their overlapping imaging features. In our case, contrast-enhanced CT revealed a well-circumscribed mass with marked hypervascularity and prominent surrounding feeding vessels. These findings led to an initial radiologic suspicion of UCD. Ideally, a preoperative needle biopsy should be considered to obtain a definitive diagnosis. However, a separate preoperative fiberoptic bronchoscopy attempted the day before surgery had to be discontinued midway. During this procedure, the patient experienced a sudden exacerbation of dyspnea and tachycardia. This acute deterioration was likely caused by the bronchoscope further occluding the critically stenotic airway (residual lumen of 4 mm), coupled with the supine position exacerbating tumor compression on the SVC and decreasing venous return. Because of this positional intolerance and the highly hypervascular tumor, performing a CT-guided or endobronchial ultrasound (EBUS)-guided biopsy was deemed dangerous. Therefore, in this life-threatening condition, the risks of any biopsy outweighed the potential benefits. The findings from the aborted bronchoscopy and pulmonary function testing were consistent with a fixed mechanical obstruction, corroborating the multidisciplinary decision to proceed directly to life-saving surgical decompression.

The patient’s severe anatomical distortions posed critical perioperative risks involving both airway and superior vena cava (SVC) management. Most notably, these include difficulty in airway management and the risk of complete potential airway collapse during induction (10–12). Furthermore, inappropriate postural adjustments can mechanically exacerbate SVC compression, severely impairing venous return and reducing right ventricular preload, and ultimately precipitate profound hemodynamic instability during the perioperative period (11–13).

To mitigate these risks, we adopted a multidisciplinary and physiologically guided perioperative strategy. Three-dimensional (3D) printed models provided spatial information beyond that obtainable from conventional imaging alone, allowing the team to anticipate the specific threats and optimize perioperative planning in advance. Given the marked hypervascularity of the tumor, preoperative embolization was performed to minimize intraoperative bleeding and allow subsequent anesthetic and surgical management to proceed under more controlled conditions. The positioning strategy was likewise grounded in the same physiologic reasoning. Initially, critical airway obstruction represented the paramount concern. Because the mass compressed the trachea from the right side, a slight right-tilted position was adopted during awake intubation. This utilized gravity to pull the tumor away from the tracheal lumen, thereby facilitating awake intubation. Furthermore, spontaneous ventilation was maintained to minimize the risk of airway collapse associated with anesthesia muscle relaxation and mechanical positive-pressure ventilation. Once the airway had been secured, the primary concern shifted from respiratory compromise to hemodynamic instability related to SVC compression. Accordingly, the patient was repositioned to a left-tilted position to improve venous return. Through dynamic physiologic optimization and multidisciplinary coordination, VATS resection was safely completed with stable intraoperative oxygenation and hemodynamics, minimal blood loss, and rapid postoperative recovery.

The immediate postoperative resolution of dyspnea and uneventful recovery further highlighted the effectiveness of this coordinated perioperative strategy. This case also underscores the value of close multidisciplinary collaboration among thoracic surgeons, anesthesiologists, and interventional radiologists in the management of mediastinal tumors with critical airway and SVC compression.

Given the achievement of complete (R0) resection and the low-grade histopathological features of the FDCS, together with rapid postoperative symptom resolution, adjuvant chemotherapy or radiotherapy was not indicated. Following multidisciplinary team (MDT) discussion, a strategy of close clinical and radiological surveillance was adopted, also in accordance with the patient’s preference.

Finally, we must acknowledge several limitations in this case report. First, due to the emergency nature of the patient’s critical airway compromise, specific preoperative serum tumor markers (such as AFP, β-HCG, and LDH) were not obtained. We recognize that these studies are clinically essential for the initial differential diagnosis to exclude germ cell tumors and lymphomas in a young male presenting with a mediastinal mass. Second, the follow-up period is relatively short. Currently, the follow-up is limited to a 5-month telephone contact, lacking structured radiological or laboratory surveillance. Although the patient self-reported being in good health, this does not equate to a true clinical cure. Therefore, continuous and strict surveillance remains mandatory for this patient.

## 4 Conclusion

In conclusion, early recognition of SVC syndrome and airway compromise, comprehensive preoperative evaluation, embolization of feeding vessels, and individualized anesthetic strategies are critical for ensuring surgical safety and favorable outcomes in patients with large mediastinal FDCS. This case emphasizes the importance of preoperative multidisciplinary discussion and interventional preparation in similarly high-risk cases. Ultimately, in selected hypervascular mediastinal masses with critical airway compromise, upfront surgery may be safer than biopsy, providing both immediate life-saving treatment and definitive diagnosis.

## Data Availability

The original contributions presented in the study are included in the article/supplementary material. Further inquiries can be directed to the corresponding author.

## Supplementary material

Supplementary Data Sheet 1 Summary of immunohistochemical and *in situ* hybridization results.

Supplementary Video 1 3D reconstruction demonstrating tumor morphology, tracheal compression, and superior vena cava involvement.
