# Supplementary material for: Case report of a giant hypervascular mediastinal mass with airway and SVC compromise managed without preoperative biopsy
Source: Front Oncol. 2026 Jun 22;16:1871556. doi: 10.3389/fonc.2026.1871556 (PMC13333509; doi:10.3389/fonc.2026.1871556)
Supplement: Supplementary Data Sheet 1 — Summary of immunohistochemical and in situ hybridization results. [file Table1.docx]

Table s1. Summary of Immunohistochemical and In Situ Hybridization Results

The marker panel confirms the diagnosis of follicular dendritic cell sarcoma (FDCS) while effectively excluding other hematolymphoid malignancies, epithelial tumors, and EBV-associated lesions.

| **Marker** | **Result** | **Diagnostic Implication** |
| --- | --- | --- |
| CD21 | Positive (+) | Primary marker confirming follicular dendritic cell origin |
| CD23 | Positive (+) | Supportive marker confirming follicular dendritic cell origin |
| Ki-67 | Positive (+, low) | Indicates low proliferative activity |
| CD20 | Negative (-) | Excludes B-cell lymphoma |
| CD3 | Negative (-) | Excludes T-cell lymphoma |
| CD138 | Negative (-) | Excludes plasma cell neoplasms |
| EBER-ISH | Negative (-) | Excludes EBV-associated tumors |
| Pan-CK | Negative (-) | Excludes epithelial tumors like thymoma or carcinoma |
